# Bioética en clave filosófica: el fundamento (bio)ontológico desde la propuesta de Juliana González Valenzuela

**DOI:** 10.18294/sc.2023.4467

**Published:** 2023-08-02

**Authors:** Roxana Nayeli Guerrero Sotelo, José Eduardo Orellana Centeno, Sabina López Toledo

**Affiliations:** 1 Doctora en Procesos Políticos. Investigadora, Instituto de Investigación sobre la Salud Pública, Universidad de la Sierra Sur, Oaxaca, México. roxanaguerrerosotelo@yahoo.com.mx Universidad de la Sierra Sur Instituto de Investigación sobre la Salud Pública Universidad de la Sierra Sur Oaxaca Mexico roxanaguerrerosotelo@yahoo.com.mx; 2 Maestro en Salud Pública. Profesor Investigador, Instituto de Investigación sobre la Salud Pública, Universidad de la Sierra Sur, Oaxaca, México. orellana17@msn.com Universidad de la Sierra Sur Instituto de Investigación sobre la Salud Pública Universidad de la Sierra Sur Oaxaca Mexico orellana17@msn.com; 3 Doctora en Biomedicina. Profesora-Investigadora, Instituto de Nutrición, Universidad de la Sierra Sur, Oaxaca, México. sabina.ltoledo@gmail.com Universidad de la Sierra Sur Instituto de Nutrición Universidad de la Sierra Sur Oaxaca Mexico sabina.ltoledo@gmail.com

**Keywords:** Bioética, Teoría Ética, Humanismo, Salud Colectiva., Bioethics, Ethical Theory, Humanism, Collective Health

## Abstract

El enriquecimiento de las propuestas bioéticas construidas desde y para Latinoamérica ha ido en aumento en las últimas décadas, a efecto de contribuir a ello es que nuestro objetivo es presentar una propuesta de interpretación bioética en clave filosófica a partir de la identificación de los conceptos centrales de la filósofa mexicana Juliana González Valenzuela. Se identificó un doble fundamento bio-ontológico: a) la “fenomenología dialéctica de la vida” que permite la síntesis entre el aspecto biológico y cultural del ser humano, así como la superación de sus contradicciones; y b) el Homo humanus que posibilita la existencia del ser bio-ético afirmado en tanto un ser auténtico y en tanto busca una vida buena (eu-bíos y eu-zoein). La reflexión y crítica de las implicaciones que el fundamento bio-ontológico apareja hipotéticamente al derecho y al poder, nos llevó a identificar las principales líneas argumentativas, pero principalmente se evidenció el vínculo necesario entre el ser bio-ético y dichas disciplinas, en virtud de su naturaleza social y comunitaria (zoon politikón, ζῷον πολῑτῐκόν).

## Abstract (en)

The enrichment of bioethical proposals built from and for Latin America has been increasing in recent decades. In order to contribute to this, our objective is to present a proposal for a bioethical interpretation from a philosophical approach based on the identification of central concepts of Mexican philosopher Juliana González Valenzuela. A dual bio-ontological foundation was identified: a) the “dialectic phenomenology of life,” which allows the synthesis between the biological and cultural aspects of human beings, as well as the overcoming of its contradictions; and b) the Homo humanus, which enables the existence of the bio-ethical being affirmed as an authentic being and one who seeks a good life (eu-bios and eu-zoein). Reflection on and critique of the implications that the bio-ontological foundation hypothetically couples to law and power led us to identify main arguments. However, the necessary link between the bio-ethical being and said disciplines was evidenced by virtue of their social and communitarian nature (zoon politikón, ζῷον πολῑτῐκόν).

## INTRODUCCIÓN

En 1978, bajo la dirección de Warren Reich, se publicó la primera Enciclopedia de Bioética la cual definía la nueva disciplina como “El estudio sistemático de la conducta humana en el campo de las ciencias de la vida y la atención de la salud, en tanto que dicha conducta se examina a la luz de los principios y valores morales”^1^. Años más tarde, Alfonso Llanos Escobar la conceptualizó como “el uso creativo del diálogo inter y transdisciplinar entre ciencias de la vida y valores morales para formular, articular y, en la medida de lo posible, resolver algunos de los problemas planteados por la investigación y la intervención sobre la vida, el medio ambiente y el planeta Tierra”^2^. Actualmente se sugiere una subclasificación de la bioética atendiendo al ámbito de acción y reflexión y es así como se afirman la bioética clínica, la teórica, la normativa, la práctica (especial o aplicada), la cultural y la institucional.

Desde el punto de vista epistemológico, el diálogo inter y transdisciplinario, así como los ámbitos de acción y reflexión generan escenarios de complejidad caracterizados por la simultaneidad de tipos de conocimiento, de los que surgen relaciones entre el conocimiento práctico, teórico y especulativo (tecnológico, científico y filosófico). Si bien cada uno de ellos tiene un sustento racional al privilegiar el acceso y significación del mundo mediante las facultades razón, lo cierto es que, la forma, las características, las normas metodológicas y el objeto del conocimiento difieren.

En las últimas décadas el desarrollo de la bioética se ha centrado principalmente sobre los conocimientos teóricos y prácticos (científicos y tecnológicos)^3^. En el primero de ellos, encontramos las propuestas realizadas desde diversas ciencias como la medicina, la enfermería, la historia, la sociología, la antropología, la etnología, entre otros. En tanto que la bioética como conocimiento práctico corresponde al mundo fáctico de la praxis en el que se establecen los valores, su jerarquía y las formas de ponderación o cálculo aplicados al proceso de toma de decisiones, por tanto, se encuentra vinculada no solo a la moral, sino también al derecho. 

No obstante, la bioética no ha tenido un desarrollo semejante en la filosofía. El conocimiento filosófico de forma genérica e independientemente de la disciplina, escuela o filósofo busca explicar las condiciones de posibilidad del ser y del mundo. En este sentido, es una reflexión crítica, profunda y continua que explica y justifica tanto la realidad como las predicaciones de verdad o de falsedad sobre ella, también trata de dilucidar el origen o primera causa al responder a la pregunta ¿por qué?^3^^,^^4^. Es por ello que en este texto nos proponemos exponer las principales ideas que justifican y explican la bioética desde la propuesta de la filósofa mexicana Juliana González Valenzuela, pues de forma única y original vincula la ética a la ontología.

## METODOLOGÍA

Para el análisis e interpretación de los textos se partió de la hermenéutica analógica propuesta por Mauricio Beuchot, en razón de que la interpretación del texto debe ubicarse en un punto medio de tal forma que oscile entre una interpretación objetivista (intención del autor) y subjetivista (intención del lector) retomando no solo la prudencia (*phrónesis*) sino la idea de virtud (*areté*) de la filosofía aristotélica. Desde el punto de vista analógico la interpretación también debe buscar una significación intermedia que medie entre lo unívoco y lo equívoco^5^.

La búsqueda y revisión documental de textos cuya autoría o coautoría perteneciera a Juliana González Valenzuela se realizó en los meses de enero a mayo del 2022. Los textos seleccionados fueron los siguientes libros: *La metafísica dialéctica de Eduardo Nicol* de 1981, *El malestar en la moral: Freud y la crisis de la ética* de 1986, *Ética y libertad* de 1989*, El héroe en el alma: tres ensayos sobre Nietzsche* de 1993, *El ethos: destino del hombre* de 1996, *El poder de Eros: Fundamentación y valores de ética y bioética* de 2000, *Genoma humano y dignidad humana* de 2005, y *Bios: El cuerpo del alma y el alma del cuerpo* de 2017.

## BIOÉTICA DESDE FILOSOFÍA

Abordar la bioética desde la filosofía implica, necesariamente, preguntar sobre sus condiciones de posibilidad, es decir, determinar las razones en las que se funda su existencia. La bioética deriva etimológicamente del griego *βιος* (*bios*) que significa vida y cualidades de existencia como son el modo, género, tiempo, duración, medios y finalidad, y de la palabra *ἦθος* (*ethos*) que significa hábito o costumbre. A su vez, la ética es una disciplina de la filosofía que justifica y explica la moral desde dos concepciones diversas: la primera, determina la esencia o naturaleza del ser humano y, a su vez, la finalidad de su conducta y los medios; en tanto, la segunda, determina las causas, razones o motivos de la conducta humana^6^. A lo anterior, se agrega la denominada metaética que tiene como fin el estudio de conceptos, métodos de justificación y supuestos ontológicos propiamente, es decir, examina la naturaleza y fundamentos de las creencias éticas^7^.

También es importante señalar que los principales problemas fundamentales de la filosofía^8^ y de la ética son retomados, y en ocasiones resignificados desde la bioética, entre ellos destacan: el problema del ser y de lo absoluto, el del conocimiento frente al escepticismo, el de la libertad frente al determinismo, y el de lo mental (ideal o abstracto) respecto de lo físico (fáctico), el del valor frente al disvalor o de lo virtuoso respecto de lo vicioso, entre otros. Consideramos que el problema fundamental de la bioética es justificar al sujeto bio-ético y sus juicios respecto al valor, y sus conceptos principales son la consciencia, la voluntad, la intención, la virtud, el bien, la justicia, el valor (bueno, útil, legal, etc.), la libertad, la responsabilidad, la obligación, el deber, la ley, la ciudad o el estado, entre otros. 

Una vez esclarecidos los campos o ámbitos de la ética, se vislumbra que algunas de las construcciones teóricas hegemónicas que definen a la bioética no logran alcanzar el nivel de profundidad precisado en filosofía como es el caso de identificar conceptos, acciones o elementos de la práctica bioética con ciertas escuelas filosóficas como son la utilitarista, la estoica, la platónica, la kantiana, la marxista, la nietzscheana, la foucaultiana, entre otras. Es decir, si bien existe un diálogo interdisciplinar, las explicaciones no son suficientes como para fundar y justificar al sujeto ético. 

También ocurre que, de existir dicha justificación filosófica de la bioética, esta no alcanza a impactar los ámbitos en los que se produce y reproduce el conocimiento bioético como son las universidades, los hospitales, los centros de investigación, las instituciones gubernamentales, las empresas privadas, entre otras. Dicha carencia resulta sumamente perjudicial, pues la naturaleza reflexiva de la ética es desvirtuada, quedando solo el ejercicio de una razón instrumental. De esta forma, la acción de razonar la bioética pasa del razonamiento a la racionalización, es decir, de la crítica al cálculo. En la razón instrumental, de acuerdo con Horkheimer, “se realiza a sí misma cuando niega su propia condición absoluta -razón con un sentido enfático- y se considera como mero instrumento^3^. 

En función de lo anterior, la bioética abordada desde la razón instrumental nos llevaría a formular y jerarquizar principios, a diseñar fórmulas o cálculos de la acción, a construir *check-list*, formularios o test estandarizados, a diseñar manuales que propician una enseñanza dogmática, entre otros, pero no nos brindaría las razones por las que se justifica y explica la necesidad del sujeto bio-ético. Para varios filósofos es sumamente preocupante la función de la razón actual, pues implica la pérdida de aquello que caracteriza esencialmente al ser humano que es la razón como ejercicio crítico^3^. Juliana González Valenzuela observó dicha deficiencia y elaboró una propuesta de fundamentación ontológica de la bioética que explicaremos a continuación. 

### Fundamentos (bio) ontológicos de la bioética

Juliana González Valenzuela, al interrogarse sobre la bioética y, específicamente, sobre su relación con el ser humano en el siglo XXI, llega a la conclusión de que tienen una relación de causalidad, al ser su fundamento ontológico, es decir, la razón que justifica su existencia y la explica se encuentra en el ser. El ser que fundamenta la bioética tiene dos movimientos recíprocos y codeterminantes: el primero, denominado *fenomenología dialéctica de la vida*, sirve para explicar el tránsito de la materia a la vida orgánica y, el segundo, denominado *Homo humanus* justifica el tránsito de la vida orgánica hacia la vida humana.

*Grosso modo*, el ser se actualiza fenomenológica y dialécticamente, lo que significa que se produce y (re)produce a través de su aparición en la conciencia, y este aparecer es dialéctico, en tanto implica que la unidad de sentido en la cual se actualiza procede de resolver una contradicción entre elementos. Es importante mencionar que si la contradicción se resuelve mediante la conciliación armoniosa deviene un “ser auténtico”, en tanto que si se descarta una de las opciones por elegir otra resulta un “ser inauténtico”, pues se funda en la negación de una de ellas. Con ello González Valenzuela trata de evitar que el fundamento ontológico caiga en movimientos reduccionistas o dualistas, pues no solo se excluye la riqueza y complejidad sino también propicia un “ser inauténtico”.

El ser auténtico *es* en tanto ético, es decir, es sujeto porque es ético: *Homo humanus*. El ser tiene por fin lo bueno, pues su pensar y reflexionar lo llevan a una vida buena o *eu-bíos* y *eu-zoein.* En este sentido, la fundamentación de la bioética es tanto ontológica como ética.

### Fenomenología dialéctica de la vida

Al reflexionar sobre el ser humano y revisar las teorías sobre el origen de la vida, Juliana González Valenzuela se pregunta sobre la metamorfosis que va de la materia hacia la vida y de la vida hacia la libertad. El flujo o movimiento se expresa como una “*continuidad discontinua*”, pues si bien hay una permanencia de elementos, rasgos o características también hay transformación; pero en el orden ontológico, el ser no tiene fracturas o fronteras absolutas, sino solo cambios en su forma. De esta manera, del *Big Bang* y de la formación de la Tierra “*se salta*” a la existencia de materia físico-química y de allí a la construcción del organismo vivo o *bíos*.

Es en este escenario en el que surge la vida orgánica del ser humano. Para Juliana González Valenzuela el *Homo sapiens* se logra con el perfeccionamiento del cuerpo (el pulgar o caminar bípedo), el desarrollo de habilidades (construcción de herramientas, dominio del fuego o agricultura) y de conocimientos (lenguaje y comunicación) por lo que implica la integración de aspectos biológicos, racionales y emocionales; no obstante, estas habilidades y conocimientos son compartidas por otras formas de vida, como los chimpancés, con las cuales hay diferencias de grado de desarrollo o de estructuración genética. De acuerdo a la filósofa, la expresión más acabada se logra con el arte, pues a lo anterior se suma la capacidad simbólica con la que se sintetizan la técnica, el conocimiento y el juicio estético-ético, dado que suelen proyectarse imágenes que representan la virtud, el bien, la justicia^10^. En este caso, el ser humano no solo actúa *(praxis*) y piensa (*nous)* sino crea (*póiesis*), en función del mundo social creado por el propio ser humano. 

De lo anterior se puede inferir la forma en que la “*continuidad discontinua”* opera en el *bios,* si bien hay una continuidad o semejanza entre animales humanos y no humanos como son los genes, las habilidades, los conocimientos, entre otros, también hay discontinuidades que permiten la diferenciación, como es la capacidad creativa o artística. En virtud de lo anterior, se logran vislumbrar más claramente dos movimientos: el primero, es la convergencia armoniosa de elementos no excluyentes sino complementarios; y, el segundo, que ese “salto”, “fractura” o “transformación” es y no es simultáneamente pues surge sobre la base de la continuidad. Es por ello que utiliza el término “emergencia”, por el cual significa la presencia de una transformación cualitativa originada por el aumento progresivo de complejidad y de integración^9^^,^^10^.

Sobre esos dos movimientos se realiza la “*fenomenología dialéctica de la vida”* pues del encuentro entre elementos contrarios se devela la complementariedad o implicación recíproca, ya que todo es unidad y continuidad. Filosóficamente implica afirmar la identidad y unidad tanto del ser como del tiempo (devenir). En este sentido, el cambio o transformación sucede en el devenir histórico, por lo que hay discontinuidad y no hay predictibilidad. Para ilustrar, utiliza tanto la biología de la evolución como la embriología, por ejemplo, un embrión es un hecho nuevo en cuanto no estaba predeterminado en sus antecedentes pero, a la vez, puede ser comprendido a partir de ellos, en suma, es un “salto sin salto” que deriva del crecimiento de la complejidad^9^.

De los supuestos de la “*fenomenología dialéctica de la vida”* es necesario concluir que la diferencia entre animal humano respecto del animal no humano solo es aparente y transitoria, en su lugar tendríamos que asumir su continuidad armónica. De lo anterior también se deriva que el ser humano es una forma más de vida orgánica entre otras. En este sentido, las relaciones de poder del ser humano y de la sociedad con respecto a otras formas de vida debe ser orientada bajo un criterio o consciencia de igualdad de origen común y no de supremacía, en oposición a la praxis ética y política hegemónica guiada por la supravaloración del ser humano y la infravaloración de otras formas de vida. Finalmente, es importante señalar que los supuestos ontológicos también impactan en la epistemología y la gnoseología, pues conllevan a reafirmar la necesidad de un conocimiento inter o transdisciplinario que permita superar las oposiciones binarias sobre las cuales se ha construido la ciencia (cientificismo).

### Homo humanus

La *fenomenología dialéctica de la vida* permite comprender el tránsito que va de la materia hacia la vida o *bíos*, en tanto el concepto de *Homo humanus*, el salto de la vida orgánica o *bíos* hacia la libertad. Juliana González Valenzuela plantea sus argumentos partiendo de una aproximación científica al ser humano denominada por Jean-Pierre Changeux como “*hombre neuronal”* quien, en el libro *La naturaleza y la norma: lo que nos hace pensar*, enfrenta sus argumentos biomédicos a los del filósofo Paul Ricoeur como a continuación exponemos.

Juliana González Valenzuela retoma el debate entre Paul Ricoeur y Jean-Pierre Changeux pues, partiendo de *L’homme neuronal* y de los avances de la epigenética, abordan el problema clásico del ser y el deber-ser desde el discurso biológico y filosófico cerebro-mente. Ricoeur señala que la contraposición entre ser-deber ser, o bien, cerebro-mente solo es aparente, como sucede con otros muchos dualismos filosóficos. En palabra de Ricoeur:

Lo que yo llamo la ética, más que la moral, con sus leyes y sus prohibiciones, está, según yo, muy arraigado en la vida, aun si no puedo evitar el momento del paso a la norma. ¿Por qué ese paso obligado? Y bien, porque la vida en su evolución nos ha dejado, en cierto modo, en proyecto: quiero decir que la organización biológica me lleva tal vez a ciertas disposiciones a la asociación, a la benevolencia; pero allí están, también, la violencia, la guerra y, por tanto, hay que encontrarnos con la prohibición, la del asesinato, la del incesto, de modo que nos encontramos en una relación de continuidad-discontinuidad, continuidad entre la vida y una ética bien arraigada en la vida, y discontinuidad al nivel de una moral que retoma en cierto modo el relevo, por cuenta propia, dado que la vida nos ha dejado en mitad del arroyo, sin darnos reglas para hacer prevalecer la paz por relación con la guerra, por relación con la violencia.^11^

Con lo anterior, se pone de relieve el problema de la continuidad-discontinuidad en la bioética, pues expresa que desde la biología se puede vislumbrar una ética arraigada a la vida; sin embargo, al mismo tiempo esta evolución ha dejado al ser humano como “proyecto”, en tanto que tiene una serie de capacidades y habilidades que le permiten cuestionar esas determinaciones biológicas. Por ejemplo, la violencia en términos biológicos es un medio para alcanzar otros fines como alimento o la supervivencia, pero la violencia en términos históricos y culturales ha sido concebida como fin en sí misma o como medio excepcional para ciertos fines. 

Desde nuestra perspectiva el denominado *cerebro plástico* o *plasticidad cerebral* sirve para visibilizar más claramente el abordaje científico y filosófico de la bioética. Este concepto “expresa la capacidad adaptativa del sistema nervioso para minimizar los efectos de las lesiones a través de modificar su propia organización estructural y funcional”^12^. El *cerebro plástico* lo podemos relacionar inferencialmente con otros dos conceptos, a saber: evolución y adaptabilidad. 

La evolución procede de la biología y entraña para Darwin la denominada selección natural e implica el cambio embriológico o de especies en función de la descendencia de un ancestro común. Dicha variación tiene su origen en la adaptación que el organismo hace respecto del medio en su lucha por la existencia^13^. De acuerdo a Rosaura Ruíz, lo determinante en la propuesta del biólogo es el carácter azaroso de la calidad de la variación^14^, es decir, hay una continuidad de las discontinuidades, y las formas en las que se materializan son impredecibles, lo que implica necesariamente la imposibilidad de afirmar una ley o una relación causal perenne desde la lógica. Aplicado al *cerebro plástico* implica que la modificación estructural y funcional puede o no presentarse en un organismo.

La adaptabilidad es un concepto propuesto desde la teoría general de sistemas y después resignificado desde la administración. Su sentido original refiere a la capacidad que tiene un sistema biológico de modificar la organización, estructura o jerarquía de sus elementos o componentes; su segundo significado implica un proceso continuado de autoorganización y aprendizaje^15^. Es con el segundo significado que el discurso biologicista se acerca al discurso social y filosófico pues implica que la adaptabilidad no es azarosa, sino es un acto esencialmente humano al implicar un acto de enseñanza-aprendizaje fruto de la voluntad, la intención y la razón. Es por ello que Juliana González Valenzuela afirma que el cerebro humano esta genéticamente determinado para la libertad^9^.

Es sobre las ideas de enseñanza-aprendizaje y de libertad que el concepto de evolución resulta insuficiente para comprender y explicar al ser humano, para ello tendrá que contemplarse el concepto de historia. La historia tiene un doble significado, por un lado, refiere tanto al conocimiento de las narraciones de hechos humanos como su sistematización; y, por otra, refiere a la totalidad de ellos (*res gestae*) en tanto significados atribuidos a la realidad histórica y ellos son principalmente cuatro, a saber: pasado, tradición, mundo histórico y sujeto historiográfico^5^. En ambos significados es patente que frente a la predeterminación se opone la libertad, manifestándose en la creación y donación de sentido que es plural y comunitaria, implica la creación de la dimensión simbólica de la realidad misma que sirve como un discurso, una práctica y un ritual que produce y re-produce la identidad y las subjetividades. 

Es esa potencia creadora manifiesta en la historia que nos permite identificar en el ser humano su naturaleza humana, pues a la mera vida biológica, genética o neuronal, denominada en griego *Zoé,* se suma la vida humana de la significación de la realidad y del ser por la cual se determinan las formas de vida llamada *bíos.* En este sentido, el ser es conjunción de *Zoé-bíos*, es decir, de vida material-vida humanizada. 

Así emergen los problemas fundamentales de la filosofía y la bioética como una disertación entre predeterminación-libertad, evolución-historia, cerebro-mente, naturaleza-cultura, cuerpo-alma, entre otros. 

Asumiendo la “*continuidad discontinua”* y frente a estos elementos no puede operar la exclusión de uno de ellos, sino reconocer su implicación recíproca que se integra de una “*sincronía vertical*” y una “*diacronía horizontal*”^9^. De esta forma, el ser humano es “*hombre neuronal”* y *“sujeto ético”* simultánea e independientemente de la perspectiva temporal. Esta forma de vincular los elementos es denominada por Juliana González Valenzuela como *racionalidad dialéctica* y caracteriza al *Homo humanus.*

El *Homo humanus* es complejidad actual de lo real e implica la integridad de los contrarios ser y no-ser, es decir, es determinado y determinante, de esta forma se encuentra determinado por la genética, por las conexiones neuronales, por los relatos fundadores de la historia, por las normas jurídicas y las costumbres, pero simultáneamente es determinante, en tanto libre posee la capacidad de autosignificarse. En este continuo devenir y movimiento entre ser y no-ser es que el ser humano va realizándose y construyéndose a sí mismo desde su pensar, decidir y actuar. Juliana González Valenzuela retomando a Freud explica que se trata de un *continuum* indiviso pero, a la vez, diferenciado que va de lo inorgánico a lo orgánico y de lo somático a lo mental^9^. Esto es uno de los grandes aportes de la filósofa a la ontología pues implica una constitución dialéctico-fenomenológica. 

La libertad del *Homo humanus* no implica una idea de ilimitación ni menos aun de irresponsabilidad; por el contrario, el ejercicio de la razón y la voluntad que llevan al sujeto a la decisión y a la acción se encuentran limitadas; y viceversa, el determinismo genético o neurobiológico no es forzoso e inevitable, sino probabilístico y más aún azaroso. Es en esta unión y simultaneidad que *bíos* es un determinante ontológico en ambos casos, que posibilita nuevas formas de pensar-actuar. De esta forma, en lugar de significar el cerebro en oposición a la mente o del cuerpo frente al alma, los podemos idear para el primer caso como un alma encarnada o cuerpo espiritualizado y, para el segundo, como una mente cerebral o un cerebro mentalizado. 

Para la filósofa, el *bíos* implica necesariamente el concepto de *ethos* que, *grosso modo*, puede comprenderse como la dialéctica de vida del *Homo humanus*, en la que se entrecruza el plano ontológico (ser) con el axiológico (valor) y los valores son un *desiderátum* de humanización, es decir, un deseo radical de humanización, en oposición a los contravalores que llevan a modos de deshumanización e inhumanidad^16^. La humanización del ser humano consiste en el trabajo continuado que lo lleva a la excelencia y a la virtud, es decir, el ser desarrolla su humanidad siendo ético. Y es con toda esta explicación del estatuto ontológico fundado en el *bíos* que podemos comprender el término con el que iniciamos nuestra disertación, a saber: el sujeto ético. Juliana González Valenzuela establece al respecto:

El misterio del hombre consiste en que es una dualidad, una materia-forma, que se hace logos, palabra, razón. El hombre es esa singular creación de la vida que logra el prodigio de autoconocerse y transformarse por medio de la mente y la mano […] El humano lleva en su ser el poder de su humanización, de hacer o construir históricamente su propia humanidad, de hacerse humano, junto con el poder de su des-humanización y de su in-humanidad; la facultad de no hacerse, de destruir su propia humanitas. Su in-determinación es la clave de su ser; su condición abierta, indefinida que le deja libre para darse a sí mismo su sitio en el mundo. Ahí radica su physis o naturaleza “esencial”, pero no “equivalente” a “esencia” (ser en sí, idéntico y absoluto). La constitución genética condiciona al hombre para autoregularse, auto-crearse. Le deja un margen de apertura; de posibilidad de ser o de no ser; de ser así o de infinitos modos: hombre-Proteo, hombre-Hamlet, hombre-Fausto, hombre-Jano; hombre-Quetzalcóatl, hombre-Centauro, Homo-humanus. Sólo el hombre puede ser humano e in-humano; sólo él puede negar su ser o afirmarlo, de ilimitadas y nunca predeterminadas maneras.^17^

El sujeto ético se humaniza *siendo virtuoso*, realizando los valores que confirman tanto la libertad como la responsabilidad. Dicha actividad no exige el ejercicio de una racionalidad instrumental sino de una racionalidad reflexiva y prudente que sintetiza lo universal y lo particular, lo corporal y lo espiritual, lo cerebral y lo mental, entre otros. El conocimiento y la praxis del sujeto ético o *Homo humanus* lo llevan a una *vida buena* o *eu-bíos* y *eu-zoein.*

### (Bio)ontología, política y derecho

La bio-ontología del *Homo humanus* busca la *vida buena* o *eu-bíos* y *eu-zoein* y lleva a aceptar la responsabilidad por el Otro, ya se trate de un ser humano, un ser no humano e incluso el mundo. Para Aristóteles *eu-zen* significa vivir bien; sin embargo, tiene dos dimensiones: una dimensión que comprende a todo ser vivo y se actualiza como un vivir bien llano y, otra, reservada al ser humano, en la que el vivir bien se entiende como vivir una vida justa, es decir, la vida es conducida conforme al fin específico del ser humano, cuya virtud más álgida es la justicia^18^^,^^19^. La vida justa es solo posible en razón del Otro, es decir, a partir de una vida colectiva, comunal o comunitaria, es por ello que la propuesta bio-ontológica conlleva, urgente y necesariamente, la problematización de la ordenación social en la que existe y vive el ser humano.

En efecto, de asumir el fundamento bio-ontológico de la vida en su doble aspecto, es decir, tanto la *fenomenología dialéctica de la vida* como el *Homo humanus*, habría la necesidad lógica de reinterpretar tanto al animal político (ζῷον πολῑτῐκόν) como al espacio social en que habita denominado polis (πόλις), pues además de existir una mutua determinación entre el sujeto y la sociedad, la polis es el espacio donde se constituye la subjetividad del ciudadano y en donde se reproduce el sentido de la vida, la jerarquía de valores, las formas de producción y consumo, las formas ordenación y de solución de conflictos, entre otras. Es importante señalar que, para Aristóteles, la polis es el lugar en el que habita el hombre por naturaleza, y en el que se transforma de un ser social (familia) a un ser político (sociedad). Es solo en la comunidad y mediante la enseñanza que realiza el ideal superior de vivir bien (*eu-zen)* y por el cual es posible la felicidad o eudaimonia (εὐδαιμονία).

El proceso anteriormente descrito implica la síntesis vital entre la cultura y la naturaleza, por la cual se constituye al sujeto bio-ético, es decir, un ser dotado de razón, voluntad e intención, que proyecta desde sí, en sí y sobre el mundo una ética arraigada a la vida. Es en la síntesis en la que el sujeto, si es consciente de su historia y su contexto, se torna un ser auténtico. Esta exigencia crítica es común a otras propuestas de bioeticistas latinoamericanos y caribeños como son las formuladas por Volnei Garrafa y Dora Porto, Juan Carlos Tealdi, Pedro Luis Sotolongo y Miguel Kottow.

El sujeto bio-ético, al ser un ser social, es también un sujeto político y jurídico, es decir, la bio-ontología se encuentra vinculada de forma necesaria tanto al poder como al derecho, pues son estos los que determinan la construcción y normalización del espacio social en los que habita el sujeto. De forma específica implican para el sujeto bio-ético:

*desde el poder*: la capacidad de obrar y producir efectos en el mundo, de influir o determinar la conducta de otros sujetos, o bien, de participar en el ejercicio mismo del poder a través de ciertas formas y contenidos; *desde el derecho*: implica que su conducta se encuentra ordenada y regulada por un conjunto de normas jurídicas creadas por una autoridad competente, y que la conducta regulada puede ser exigida a través del uso legítimo de la fuerza pública dado su carácter vinculante y coercitivo.

Problematizar al sujeto bio-ético desde el poder y el derecho no es una tarea fácil, ya Bobbio adelantaba la relación paradójica que mantienen ambos términos, pues son dos caras de la misma moneda, donde el poder sin derecho es ciego y el derecho sin poder queda vacío^20^. La relación de interdependencia y codeterminación entre poder-derecho explica por una parte el paso del poder de hecho (*de facto)* al poder de derecho (*de iure)* mediante el cumplimiento del principio de legalidad y legitimidad y, por otra, la diferencia entre una norma válida y una norma eficaz, a través del principio de efectividad. En suma: el poder precisa de su ordenación, control y aceptación que legitime el discurso y las acciones de la autoridad, en tanto que el derecho para ser eficaz precisa de la fuerza y la coacción de la autoridad a efecto de hacer cumplir las normas jurídicas. 

Partiendo de estas relaciones complejas que vinculan poder-derecho y retomando el estatuto de la bio-ontología podemos señalar una novedad radical: hay una doble integración de la vida determinada por la naturaleza y la cultura, de esta forma la potencialidad creadora (y destructora) de la libertad humana se haya limitada, desde un inicio, por la responsabilidad originaria por el Otro, cristalizado en una pluralidad de formas de vida y no solo el prójimo humano, es decir, no existe una jerarquización ontológica. Esta máxima ética de la bio-ontología implicaría para el derecho una “esfera de indecidibilidad”^21^, y para la política un “territorio o frontera inviolable”^22^ o “coto vedado” como explicaremos a continuación.

La “esfera de lo indecidible” caracteriza a las constituciones rígidas y se traduce en la categoría jurídica de validez sustancial, pues implica sustraer de la decisión de la mayoría la violación de ciertos principios (absoluta), o bien, imponer ciertos procedimientos extraordinarios (relativa). Los principios a los que refiere Ferrajoli son eminentemente jurídicos y son aquellos que estructuralmente definen actualmente a las democracias constitucionales, es decir, los que incumben a los límites y vínculos impuestos tanto a los poderes públicos como a los poderes privados. En este sentido, los “poderes absolutos y salvajes” del mercado, de las telecomunicaciones, de las farmacéuticas, de las empresas trasnacionales, de la agroindustria, entre otras, son limitados y controlados a efecto de garantizar y proteger los “derechos de los más débiles”. 

En la bioética ¿existen temas, tópicos o contenidos sustraídos a la decisión de los poderes públicos y garantizados frente a la fuerza de los poderes privados? No hay un consenso entre los doctrinarios por lo que hace al contenido y extensión de los significados; sin embargo, si coinciden en que los principios generados en la bioética son insuficientes por sí mismos para regular jurídicamente el fenómeno, independientemente que se trate de los principios bioéticos del principalismo, los principios bioéticos del personalismo, los principios de acción basados en la evidencia de Cochrane, o bien, los seis objetivos de la medicina según el Institute of Medicine de EEUU. Entre los teóricos destacan Manuel Atienza, Eduardo Luis Tinant, Ernesto J. Vidal Gil, Erick Valdés y Laura Victoria Puentes, Eduardo Rivera López, entre otros.

Por otra parte, el fenómeno de la globalización ha propiciado y visibilizado el pluralismo de fuentes jurídicas. Así encontramos la coexistencia de ordenamientos normativos prescriptivos y coercitivos del *hard law* -como son, en el caso de México, la Constitución, la Ley General de Salud, entre otras- y de ordenamientos voluntarios y no coercitivos del *soft law* como pudieran ser las pautas de las Asociación Médica Mundial (AMM) y del Consejo de Organizaciones Internacionales de Ciencias Médicas (CIOMS)^23^.

Dicha integración jurídica obedece a la propia naturaleza de la bioética, la cual además de ser inter o transdisciplinar, también es heterogénea y compleja. El mismo fenómeno desde la epistemología jurídica y la teoría de la argumentación jurídica solo es compatible con el giro sistémico del pluralismo jurídico fundamentado en la teoría general de sistemas, pues permite la articulación de diversos sistemas normativos. Ejemplos de este desarrollo epistémico son las propuestas del transconstitucionalismo de Carlos Neves, el constitucionalismo transférico de Luis Claudio Araujo, el constitucionalismo multinivel de Ingolf Pernice y el constitucionalismo internacionalizado de Siddharta Legale.

Sumado a lo anterior, es preciso recordar la especial naturaleza jurídica que posee todo acto médico-sanitario, pues se caracteriza por la obligación de los profesionales de la salud de aplicar la *lex artis ad hoc* o *estado del arte médico*, es decir:

...el conjunto de normas o criterios valorativos que el médico, en posesión de conocimientos, habilidades y destrezas, debe aplicar diligentemente en la situación concreta de un enfermo y que han sido universalmente aceptados por sus pares. Esto es, los profesionales de la salud han de decidir cuáles de esas normas, procedimientos y conocimientos adquiridos en el estudio y la práctica, son aplicables al paciente cuya salud les ha sido encomendada, comprometiéndose únicamente a emplear todos los recursos que tengan a su disposición, sin garantizar un resultado final curativo.^24^

Jurídicamente, la Suprema Corte de Justicia de la Nación de México ha interpretado lo anterior como una obligación extracontractual, en el sentido de que los profesionales de la salud están obligados a actuar de acuerdo a los estándares de su profesión y de acuerdo a las disposiciones reglamentarias, principios científicos y éticos^25^, como son las Normas Oficiales Mexicanas, las Guías de Práctica Clínica, los Manuales de Procedimientos, la Clasificación Internacional de Procedimientos Médicos, el *Health Level 7*, entre otros^26^. En este sentido, la responsabilidad de los servidores públicos en el área de la salud pública no solo se origina por la inobservancia de las normas jurídicas sino también por el incumplimiento de las prescripciones de la ciencia médica, esto es, por no sujetarse a las técnicas médicas o científicas exigibles para dichos servidores -*lex artis ad hoc*-, o al deber de actuar con la diligencia que exige la *lex artis*^27^. Finalmente, es necesario subrayar que para la aplicación de la *lex artis ad hoc* debe considerarse la variabilidad y complejidad del acto médico-sanitario como son el cuadro clínico, información incompleta, infraestructura, entre otras. En este sentido, es imposible aplicar la misma normativa a todos los casos, sino que estas deben adecuarse al caso concreto:

La medicina no es una ciencia exacta, por lo que no puede pronosticar ni asegurar resultados favorables en todos los casos, dado que hay limitaciones propias del profesional en la interpretación de los hechos, como cuando el cuadro clínico no se manifiesta completamente, el paciente no comprende los riesgos y beneficios de un procedimiento diagnóstico o terapéutico, o entrega información incompleta de sus síntomas; además, las circunstancias en que se da una relación clínica pueden limitar la certeza del diagnóstico y la eficacia de medidas terapéuticas. En estas condiciones, dada la gran variabilidad y complejidad que rodean a una condición clínica concreta, algunas dependientes del profesional, otras de las condiciones particulares del paciente, de los recursos o infraestructura que se disponga y, finalmente, por las circunstancias que la rodean, es imposible aplicar la misma normativa en todos los casos, sino que éstas deben adecuarse al caso concreto. Por tanto, puede decirse que la *lex artis ad hoc* es un concepto jurídico indeterminado que debe establecerse en cada caso, en el que el médico, a través de un proceso de deliberación, aplica las medidas con prudencia a la situación clínica concreta y en la medida de las condiciones reinantes. En la órbita del derecho comparado, la Sala de lo Civil del Tribunal Supremo español ha delineado paulatinamente el referido término hasta definirlo como “aquel criterio valorativo de la corrección del concreto acto médico ejecutado por el profesional de la medicina-ciencia o arte médico que tiene en cuenta las especiales características de su autor, de la profesión, de la complejidad y trascendencia vital del paciente y, en su caso, de la influencia en otros factores endógenos -estado e intervención del enfermo, de sus familiares, o de la misma organización sanitaria-, para calificar dicho acto de conforme o no con la técnica normal requerida (derivando de ello tanto el acervo de exigencias o requisitos de legitimación o actuación lícita, de la correspondiente eficacia de los servicios prestados y, en particular, de la posible responsabilidad de su autor/médico por el resultado de su intervención o acto médico ejecutado).^28^

En función de lo anterior, resulta evidente que la interpretación y aplicación del derecho relativo a la bioética es diferente a la interpretación jurídica tradicional; en este sentido, diversos teóricos han formulado diversas propuestas teóricas entre las que destacan tanto la de Erick Valdés y Laura Victoria Puentes como las de Manuel Atienza y Eduardo Rivera López.

Ahora bien, desde la filosofía política el “territorio o frontera inviolable” y el “coto vedado” refieren las “precondiciones lógicas” de la democracia, que son identificadas por Bobbio y Garzón con ciertos derechos de libertad y, por tanto, con la tradición política liberal. A efecto de asegurar la permanencia de estos derechos y evitar cualquier limitación o supresión, se establece una línea fronteriza que la decisión política (individual o colectiva) no puede traspasar, aun cuando le asista la fuerza del principio mayoritario en razón de que son inalienables a los seres humanos^22^. De acuerdo a Bovero, se pueden identificar ciertas regiones con el “coto vedado” a saber: a) derechos de libertad personal, de opinión, reunión y asociación, b) derechos sociales de educación y subsistencia; c) principios políticos de legalidad y de imparcialidad, y d) prohibición de un poder absoluto y, por tanto, separación del poder político (control de los medios de coacción), poder económico (control de recursos y bienes) y poder ideológico (control de las ideas y del conocimiento a través de los medios de información y persuasión).^29^

De forma análoga tanto el sujeto bio-ético como el conocimiento y la praxis de la bioética se construyen y reproducen en el espacio social, tanto en el ámbito público como el privado y, en este sentido, se vinculan con el ejercicio del poder y las prácticas democráticas. Las precondiciones lógicas en el discurso y la praxis del sujeto bio-ético y de la bioética pueden identificarse de la misma forma con: derechos, obligaciones, libertades, transparencia y empoderamiento. Los procesos comunes a través de los cuales se actualizan son: el consentimiento informado y sus variantes; la participación de la comunidad y de actores sociales en las investigaciones biomédicas; prácticas inclusivas en los procesos de deliberación y de toma de decisiones; la transparencia, la solicitud de información pública y la rendición de cuentas; la inclusión y comprensión de perspectivas éticas y morales heterogéneas; el conflicto de intereses; entre otras. 

## CONCLUSIONES

La propuesta bio-ontológica de Juliana González Valenzuela tiene elementos originales que permiten dotar a la bioética contemporánea de una sólida fundamentación filosófica que explica y justifica al ser humano. La originalidad consiste en la integración dialéctica de elementos contrarios, que se manifiesta en dos movimientos: la *fenomenología dialéctica de la vida* y el *Homo humanus*. 

La bio-ontología permite superar las escisiones que históricamente han llevado al conocimiento filosófico, científico y tecnológico a operar bajo la exclusión de uno de los elementos, dando como resultado prácticas epistémicas caracterizadas por el monismo y el reduccionismo. Es por la misma razón que la propuesta es absolutamente adecuada para la bioética, en tanto se caracteriza por la inter o transdisciplinariedad y permite tanto la comprensión como la aplicación de una dialéctica que integra lo particular con lo universal.

Coincidimos con Adolfo Sánchez Vázquez al caracterizar a la propuesta de Juliana González Valenzuela como una *ética humanista,* pues de acuerdo al filósofo “hay moral porque el hombre está configurado de tal manera -como ser libre- que no puede dejar de ser moral… (en este sentido) la moral es justamente una necesidad impuesta por su libertad”^30^. En síntesis, el sujeto y su dignidad están determinadas moralmente desde su libertad.

Es preciso señalar que los ejercicios individuales o sociales de la libertad conforman modos de ser histórico, lo que lleva a concluir la coexistencia de una pluralidad de moralidades, que son estudiadas y comparadas desde la antropología, la etnografía, la historia, entre otros saberes científicos. Desde la filosofía es posible encontrar en esa pluralidad una unidad que permite la conciliación y superación tanto de diferencias como de contrariedades y, desde la propuesta de la autora, estará dado por el fundamento bio-ontológico.

Aplicando las tesis bio-ontológicas al derecho -más específicamente a la filósofa del derecho- tendríamos que asumir, como sugiere Juliana González Valenzuela, un *continuum* indiviso procedente de la relación dialéctica de continuidad-discontinuidad^10^, es decir, una relación codeterminante entre mundo del ser (naturaleza) y del deber ser (cultura) de esta forma el aspecto biológico del ser humano determina y condiciona su ser; pero, simultáneamente, ese ser determina y condiciona su existencia biológica, con lo que se actualiza la su relación dialéctica. 

En función de lo anterior, tendrían que ser analizadas y resignificadas las aproximaciones axiológicas y teleológicas de la norma jurídica por dos razones: la primera, es que las principales propuestas teóricas que definen y jerarquizan los valores son desarrolladas desde la oposición de valores como son justicia-injusticia, utilidad-inutilidad, bien-mal, virtud-vicio, individual-colectivo, entre otras, y la propuesta bio-ontológica se centra en la superación armoniosa de esas dualidades. La segunda, es que la predisposición biológica, de acuerdo a la propuesta bio-ontológica, impele al ser humano una responsabilidad no solo frente a su especie, sino también respecto a los seres no humanos y frente al mundo. En este sentido, consideramos que la comprensión del sujeto es sumamente original pues se es libre en tanto se está humanizando el ser, es decir, mientras está siendo éticamente. 

Finalmente, es de señalar que el fundamento bio-ontológico precisa analizar y problematizar la filosofía política, pues no solo justifica la consolidación de la sociedad, del Estado y su ley, sino también teoriza los criterios que determinan al poder soberano, sus fines y formas. La bio-ontología del *Homo humanus* busca como fin de la vida política del ciudadano la *vida buena* o *eu-bíos* y *eu-zoein*, que están determinados por la aceptación de la responsabilidad por el Otro (ser humano, no humano y mundo).
